# Factors influencing sport development among women with disabilities: a case study of a visually impaired Spanish Paralympic woman

**DOI:** 10.3389/fspor.2024.1518489

**Published:** 2025-01-08

**Authors:** Alejandro Barrera-Garcimartín, Áurea Redondo-Fernández, Javier Pérez-Tejero

**Affiliations:** ^1^ ‘Fundación Sanitas’ Chair for Inclusive Sport Studies (CEDI), Sports Department, Sports and Training Research Group, Faculty of Physical Activity and Sport Sciences-INEF, Universidad Politécnica de Madrid, Madrid, Spain; ^2^ ‘Fundación Sanitas’ Chair for Inclusive Sport Studies (CEDI), Department of Health and Human Performance, AFIPE Research Group, Faculty of Physical Activity and Sport Sciences-INEF, Universidad Politécnica de Madrid, Madrid, Spain

**Keywords:** Paralympic sport, talent identification, initiation, barriers, facilitators, initiatives

## Abstract

**Introduction:**

Women with disabilities may experience particular difficulties in starting and developing in sport, also in the Paralympic context. Although a great deal of research has been conducted with high-performance athletes, relatively few studies have focused on athletes with disabilities, especially Paralympic women using person-first approaches. Thus, the main objective of this study is to understand, through the experience and opinion of a Paralympian female athlete, how these athletes reach their full potential, identifying the elements and initiatives that can influence (whether positively or negatively) their sporting trajectory and developmental milestones.

**Methodology:**

This study analyzes the case of a quadruple Paralympic medalist in blind Judo, who has competed in Paralympic Games from Athens 2004 to Paris 2024. Information was gathered from a semi-structured interview, a book chapter she had written, and her participation in a forum.

**Results:**

The athlete differentiated between two key stages in the sports career of people with disabilities: (1) the beginning of sports practice; and (2) development processes and the search for future Paralympic athletes. Women with disabilities face particular difficulties in accessing sport, with foundations for people with disabilities and coaches playing a fundamental role. It is crucial to give visibility and have sports references in these areas, highlighting initiatives such as Paralympic School Days and inclusive competitions. There are conditioning factors in the identification and development of talent, such as the rules and the nature of each Paralympic sport, and the level of inclusion that the athletes have experienced in their environment.

**Conclusions:**

This case study presents the perceptions of a female athlete with regard to the beginnings and development of sport in the Paralympic framework, underlining the need to promote actions that help and encourage the development and participation of women with disabilities in sport.

## 1 Introduction

It is currently estimated that 16% of the global population lives with a significant disability. This percentage is expected to increase in the coming years (1). Physical exercise is a crucial pillar for achieving and sustaining an optimal state of health, as it plays an essential role in the maintenance and improvement of overall well-being (2). However, many people with disabilities face significant barriers that hinder their participation in physical activities (3). In this regard, it has been observed that people with disabilities are between 16% and 62% less likely to meet the recommended levels of physical activity than general population (4).

At both the international and national levels, various strategies are being developed to increase inclusion and achieve the Sustainable Development Goals for people with disabilities. Internationally, the United Nations Charter stands out because it lays the foundations for the inclusion of people with disabilities (5). In Spain, Article 6 of the current Sports Law 19/2022 establishes policies that seek to guarantee social inclusion and implement programs through sports federations and entities to raise awareness and promote inclusive sports activities (6).

In Spain, 58.6% of people with disabilities are women (7). Generally, women with disabilities perceive greater difficulties in engaging in sports than their male counterparts (8), leading to lower participation rates and levels of physical activity compared to men with disabilities (9). These limitations can be observed in the number of federative licenses among people with disabilities in Spain, where for every three male licenses, there is only one female license (10).

Participation in various physical and sports activities does not depend solely on women with disabilities themselves; it is also necessary to analyze the contextual factors (both barriers and facilitators) they face. Through the International Classification of Functioning, Disability, and Health (ICF), disability can be understood as a dynamic and psychosocial concept resulting from the interaction between impairments, activity limitations, participation restrictions, and the influence of environmental and personal factors (11). Women with disabilities have to contend with various social, physical, or psychological adversities to a disproportionate extent (12). Such a situation has necessitated the adoption of actions to mitigate this issue, as reflected in the current Sports Law 19/2022, which states:

According to Article 49 of the Spanish Constitution, the General Administration of the State, in collaboration with other Public Administrations, will promote the necessary policies to guarantee the full autonomy, social inclusion, and equal opportunities of people with disabilities in the field of sports, paying particular attention to the specific needs of women and girls with disabilities and eliminating obstacles that hinder their full integration. (Boletín Oficial del Estado, 2022, p. 193324)

These measures also aim to affect sports and competitive fields. Accordingly, many researchers have long examined the identification and development of sports talents across a range of contexts. As previous research has shown, identifying the qualities at an early age that predict future performance has proven challenging (13). Talent identification research has tended to select athletes based on their physical and psychological characteristics (14). Through considering all the possible components that most significantly influence athletes’ trajectories, a series of factors known as primary factors have been established, although some may have greater relevance than others. According to Baker and Horton, the most important elements are psychological factors, genetics, and training (15).

This research has mainly addressed athletes without disabilities. The existing lack of studies in Paralympic contexts poses a significant obstacle in the identification and development of Paralympic athletes. Although there may be some similarities between athletes with and without disabilities, there are also certain distinguishing elements between the two sport developmental processes (16). Studies related to disability incorporate differentiating aspects in these fields: Paralympic athletes’ initiation into their sport can vary depending on when they acquired their disability, which, in turn, becomes a key element in their career (17). Some research has highlighted the type and severity of the athlete's impairment and their potential functional classification in competition as a key indicator for identifying and predicting their level of success in their sport (18), as variations in the type of impairment seem to influence performance (16). A paucity of resources in Paralympic sports due to relatively lower funding can have a significant impact on these athletes, too (18).

Recently, efforts have been made to explore the factors influencing the training processes of Paralympic women in Spain, identifying different key elements in their development, including their coach, their immediate social environment, and psychological, physical, and technical-tactical aspects (19). However, the relevant literature remains scarce, with a lack of proposals for practical transfer. It is essential to understand the opinions and experiences of Paralympic athletes themselves through first-person studies to better understand the key components associated with their initial sports practice, talent identification, and development in Paralympic contexts. This research is based on the need to involve athletes with disabilities in order to elaborate and develop policies and proposals for improvement in these areas. To achieve this, it is essential to incorporate their experiences and perspectives, valuing the experiences of those who are directly involved in these realities (20).

Given the above, the primary objective of this study is to understand how a female Paralympic athlete reaches her maximum potential, and to identify the elements that can influence (whether positively or negatively) her sports trajectory from her first-person perspective. Having analyzed these perceptions, the study's secondary objectives are to: (a) identify the main barriers and facilitators that condition the initiation and development of sports among women with disabilities; and (b) to analyze what factors and initiatives can influence the development of sport for women with disabilities and the development of talent in Paralympic contexts, as is the case of the participant in this case study.

## 2 Materials and methods

### 2.1 Participant

Presented here is a case study centered on a quadruple Paralympic medalist. The athlete was born with a severe visual impairment due to complete oculocutaneous albinism and competes in the B3 functional classification for judo. She has won two silver medals and one bronze medal at the Paralympic Games, in addition to several medals at world and European championships. At the time of the research, the participant was 45 years old, had 26 years of experience in her sport, and was still active, aiming to qualify for the Paris 2024 Paralympic Games, where she ultimately achieved a bronze medal.

The participant was selected for several reasons. First, she has extensive insight into the Paralympic context due to her substantial experience in this field as an athlete. As a woman with a disability, she is fully familiar with the difficulties and obstacles that this group faces in initiating and developing sports practice. Additionally, her active commitment to promoting inclusion reinforces her suitability for this selection. Through more than 40 conferences at educational centers to date, she has shared her experiences and lessons to raise awareness about inclusion. All experimental procedures were communicated to the participant and applied in accordance with the Declaration of Helsinki.

### 2.2 Data collection and analysis

The information collected for this study was obtained from various sources to achieve a complete understanding of the topic in question. The first source of information consisted of a semi-structured interview to explore the participant's experience and perspective on the barriers, facilitators and initiatives that influence the development of Paralympic athletes. The interview topics were based on previous research in this area (21). The script was organized into four sections: the first was an introduction and warm-up questions; the second section collected the athlete's experiences throughout her career; the third addressed what the participant considered indicators of talent in the identification and development of athletes; and the final section asked the athlete to evaluate the current talent identification and detection program for Paralympic athletes and outline her ideal program in this field.

The interview was conducted online through the Zoom platform and lasted 47 min. Prior to the interview, the participant received an oral explanation of the study's purpose and was reminded of the voluntary nature of the research, with the assurance that she could withdraw at any time without any consequences. Permission to record the interview was requested subsequently.

To complement the study, participant contributions to the First Forum on Women, Sports, and Disability—MUDIDI (22) were collected. One of the forum's purposes is to analyze the barriers hindering the sports participation of women with disabilities, and thereby promote specific actions to foster their inclusion on equal terms. The athlete under study attended the forum as a representative of the Spanish Paralympic Committee, addressing these issues from her perspective and experience as an athlete and educator. Additionally, information was obtained from the book *Deporte inclusivo: aplicaciones prácticas* (*Inclusive Sport: Practical Applications*) (23), in which the participant had written a chapter on competition and inclusive judo. This document provided insights into the relevance of sport, specifically judo, as a tool for inclusion and sports practice.

For the data analysis from the three sources of information, first the interview and the forum were transcribed into a Word® document. Once transcribed, the same process of analysis was carried out with the three sources of information: the interview, the forum and the book chapter. Firstly, the coding process, a comprehensive reading was performed to gain familiarity with the data and determine the units of analysis from the interview and forum transcriptions, as well as the text extracted from the book chapter. Subsequently, preliminary labeling was performed, locating all units of analysis obtained through an open and inductive coding system. The total number of coded units of analysis was 89. Next, a branching node or axial coding system was developed using the constant comparison method (24), until stability was achieved and consensus was reached on all themes and dimensions. To mitigate any potential bias and ensure the validity of the results, the research team held multiple meetings using the triangulation or validation method (25). The verbatim is labeled according to the source of information from which it was obtained, differentiating between the book (B), the forum (F) and the interview (I). The coding and categorization process was carried out using the Word®.

## 3 Results and discussion

This research aimed to explore the factors that can influence an athlete's journey from the beginning of their sports career to becoming a Paralympic athlete. One of the secondary objectives was to analyze the various actions and initiatives currently being developed to support the processes of talent identification and detection, as well as these athletes’ development.

The research was designed as a case study due to the distinctive characteristics of the selected athlete, in order to explore and understand her opinions and insights on this topic in particular depth. It is important to highlight the uniqueness and attributes of the interviewee: (1) an athlete who has participated in multiple Paralympic Games; (2) currently active; (3) a coach; (4) pursuing an academic career parallel to her sports development (dual career); (5) involved in promoting Paralympic sports and inclusion.

The main results show the impact of the environment and various factors (for instance, lack of female role models, the coach's role, the role of foundations) on the initiation of sports practice. Additionally, from the participant's perspective, several initiatives aimed at detecting potential sports talents can be identified. Thus, the results can broadly be divided into two groups: (a) the beginnings and determinants of sports practice for people with disabilities; and (b) the development and search for future Paralympic athletes (see Table 1). This thematic structure will be followed below in order to present and discuss the results.

**Table 1:** Grouping of the obtained results.

Beginnings and determinants of sports practice	The development and search for future Paralympic athletes
– Environment (16) – Gender (4) – Clubs, federations and foundations (6) – Family (3) – Coach (9)	– Challenges (10) – External Factors (23) – Strategies (18)

The recording units are listed in parentheses to the right of each grouping.

### 3.1 The beginnings and determinants of sports practice for people with disabilities

#### 3.1.1 The environment

People with disabilities face numerous barriers to accessing environments that might facilitate their entry into sports practice. These obstacles are very diverse, comprising accessibility, economic, political, caregiving and social factors (26). Previous research has highlighted a lack of support and disapproval by others as a significant limitation (27), which can shape individuals’ participation in sports and social activities and in turn greatly affect emotional factors (28), harming their self-esteem and motivation (29), as the athlete describes:

All of this inevitably led me to a group of people with disabilities who are excluded, with low self-esteem, and assuming that this situation is the reality that applies to everything. In simple terms, it is what it is. (I).

#### 3.1.2 Gender

It is important to differentiate types of barriers according to sex. As highlighted previously, having a disability can hinder access to sports practice, and being a woman with a disability can complicate these processes further (30). In fact, in Spain, the number of sports licenses for women in federations for people with disabilities is three times lower than that for men (10). The results of this research confirm these data, clearly revealing the disproportionate difficulty women with disabilities face in participating in sports activities.

The social difficulties and limitations for women in accessing sports are quite evident. When these women have a disability, everything becomes much more complicated. (F).

Psychological factors such as motivation and fear can act as barriers to sports practice among this group (31). For this reason, social support plays a fundamental role in this area (32). Women often receive less support from family and peers than their male counterparts (33), which, combined with female athletes with disabilities’ lack of visibility, renders sports practice particularly challenging:

There is always a lack of role models. As I mentioned before, they are still lacking, and it is not that they do not exist, it is that we don’t see them. Occasionally, one appears; of course, there are many phenomenal women, but you don’t hear about them until you get into the Paralympic sphere. (F).

#### 3.1.3 The role of clubs, federations, and foundations

Clubs and federations play a crucial role in both the initiation and development of athletes. According to DeLuca's multidisciplinary inclusion framework, four approaches to inclusivity can be distinguished: normative, integrative, dialogical, and transgressive. Based on the history and references provided by the interviewed athlete, the model that most closely aligns with the current context in Spain is the normative approach, according to which minority groups are accepted only so long as they conform to the prevailing norms (34).

In some cases, clubs have implemented changes to accommodate diversity, but a predominant cultural norm persists that continues to exclude young people with disabilities. These findings are consistent with Jeanes et al.'s study in Australia, which noted that young people with disabilities remain excluded from community sports due to various reasons at club level and differing perceptions of inclusion (35). Nevertheless, some federations are currently undertaking initiatives and organizing events aimed at advancing an integrative or dialogical approach, which involves formal modifications (34). An example of this is the Spanish Federation of Judo (FEDC):

FEDC has organized adapted competitions in which athletes with and without disabilities compete together. It was the sighted judokas who adapted to the rules of judo for the blind and visually impaired. (B).

Other studies have highlighted the challenges in promoting inclusion policies, emphasizing the need for people who are willing to invest time and effort in managing and implementing these changes (35, 36). In this context, the new Law 39/2022 on Sports in Spain is noteworthy, as it stresses both the role of federations in promoting sports for people with disabilities, particularly women, and the importance of inclusive environments in sports.

At this point, foundations play a significant role. These organizations provide substantial support for people with disabilities, especially in the early stages of sports practice. Through these entities, it is possible to more efficiently promote sports disciplines and adapt them to the specific needs of each person with a disability. An example in Spain is the ONCE Foundation, the disability service provider and representative for the visual impaired and an organization that promotes social inclusion for people with disabilities, predominantly those with visual impairments. As reflected by the participant, who, prior to committing to judo, had explored various sports practices but was compelled to abandon them due to a lack of resources for adaptation:

At that time, I was not affiliated with ONCE, so we did not have a clear understanding of the exact problem to solve. (I).

#### 3.1.4 Family involvement

The role of the family is a significant topic in talent development research. According to the stages of expertise development defined by Bloom and Côté, family involvement is evident throughout the sport development process, although it is particularly important in the initial stage, the beginnings (37, 38). The same is true in the Paralympic context, as the family plays a critical role in the early years of an athlete's development (17), especially in the case of Paralympic athletes:

I believe that the family is fundamental because people—and especially children with disabilities—often depend on their parents to participate in sports due to accessibility issues. (I).

On the other hand, overprotectiveness from parents or caregivers can limit athletes’ opportunities to develop their autonomy and can lead to a loss of decision-making ability, potentially resulting in sports abandonment (39). Therefore, coaches have highlighted the importance of independence so that athletes can undergo optimal development in the Paralympic context (40).

It should be noted that many athletes, including the athlete interviewed, have not had significant positive experiences in physical education. For this reason, some athletes are forced to seek other environments to develop their sports skills, rendering family support crucial (41). A family sports history, where parents and siblings participate in sports, can spark an interest in sports participation for people with disabilities (42).

When I started college, I met people who did judo. I had always been attracted to that sport because my brother had practiced it and I knew it existed. (I).

As the athlete develops, the role of the parents also evolves. Parental influence can vary depending on the age at which the athlete begins sport practice. Although this aspect has been extensively researched and is considered highly important in athletes’ growth (36, 37), some athletes progress without being significantly affected by this factor.

Well, my family experience has not really been any, because I started as an adult and was already independent from my family. (I).

#### 3.1.5 Coach

The relationship between a coach and their athlete can be described as one that is continuously evolving and transforming, dynamic and changing. Similarly to the family context, Bloom and Côté have observed that the coach's role varies depending on the stage of the athlete (37, 38). In the initial stage (the beginning of sports practice), the climate created by the coach is crucial for the athlete's continued development in the sport. Therefore, the creation of an appropriate motivational environment is essential in promoting commitment to sport (43). The environment perceived by peers and the coach's support in building autonomy significantly affect sports motivation (44). In the Paralympic context, the influence of the environment and interaction with people with disabilities can prove particularly important in the early stages.

(He (my coach) did not assume that you would perform better or worse due to your disability, and with a level of performance demand, without being condescending, which is very typical. (I).

According to the interviewee, during the second phase (the specialization phase; (37, 38), having a supportive environment remains crucial for the athlete's development. In addition, coaches play a crucial role in supporting and developing athletes. These coaches need specific knowledge about disabilities so that they can offer training programs that are suitable and adapted to their athletes’ needs (18). To fulfill this role, coaches require a support structure that provides opportunities for specialized training, learning, and development in the field of disability (45), which they often lack. The athlete evaluated the coach's role in these early stages as follows:

I think the coach has vital, enormous importance. Let’s say capital. On a scale from 1 to 10, an 8.5, leaving some room for others to be important too. (I).

### 3.2 The development and search for future Paralympic athletes

Currently, multiple programs have been developed with the primary goal of identifying and detecting talents, especially among athletes without disabilities. These initiatives encounter a significant challenge, as athlete development often does not follow a linear path and is subject to variations (46). Numerous factors affect these processes, which are unique to each individual's sports development.

#### 3.2.1 Challenges

The search for future Paralympic athletes is a complex and challenging process. Sociocultural barriers imposed on people with disabilities can influence their participation in sports (47). A lack of fully accessible environments and the limited availability of inclusive activities or Paralympic sports pose significant obstacles to this group's participation in sports. A scarcity of information on the whereabouts of individuals with disabilities, combined with a lack of awareness about their sports options, are major issues that can limit opportunities to participate in Paralympic sports. It has been observed that relatively low levels of physical activity may correlate with a lack of awareness of sports opportunities (32):

It is important to reach people with disabilities to let them know that there is a possibility to participate in sports, because when I started judo, I did not know that judo for the blind existed. (I).

One of the main difficulties in this respect is a lack of funding (48). Media coverage is an associated issue as Paralympic athletes enjoy only limited visibility (49). Women face particular challenges in obtaining sponsorships (50), making it essential to improve the dissemination of information to increase female participation in Paralympic disciplines (51):

It is important that these structures work in a coordinated way, that the media supports us by providing an equal view of sport, and that there are role models for women with disabilities, as they are currently scarce. (F).

Regarding sports and competitive arenas, women encounter more obstacles than men (52). One of the most common challenges is the existing disparity in access to resources and opportunities, whereby women with disabilities are underrepresented in high-performance sports:

At the moment, it (the opportunities for women with disabilities in high performance) is scarce, insufficient and limiting. It is only when they reach high-performance sports, at the elite level, that the Paralympic Committee takes notice of these women and realizes that they are not reaching the level. The percentage of women (in high performance) is very small, the sports modalities are fewer, and although gender equality perspective has been adopted within the Paralympic Committee is complete, the path is not. (F).

#### 3.2.2 External factors

Eligibility and classification systems in disability sports are essential for the development of Paralympic sports. These systems have been identified as a decisive factor in the development and progression of athletes at all stages of their sporting careers, particularly during the initiation phase. It has been observed that athletes’ trajectories can vary depending on their sport class (45). Consequently, classification can have a significant impact on athletes’ success. As a result, the potential classification of athletes has been established as a key indicator in the identification and development of athletes (16).

In addition to athletes’ potential classification, the present research has identified a new factor that may be crucial in determining whether an athlete can compete at a high-performance level: the specific rules of each sport. It has previously been pointed out how the Paralympic movement itself, despite having emerged as a response to exclusion, can perpetuate forms of exclusion. Some athletes, such as those with intellectual disabilities, may be left out of the participation system due to the established classification criteria (53). However, paradoxically, some athletes may be excluded from a competition even if they have an appropriate classification, creating even a greater exclusion:

If you have a hypotonic paralyzed arm, you might not be able to swim. Your impairment will condition you, and regardless of your classification, that sport may already be barred for you as a competitor. (I).

In the Paralympic context, a significant factor is the timing of when athletes acquire their disability. It has been noted that athletes with congenital or early-acquired disabilities tend to achieve superior results more quickly, while those who acquire disabilities later progress at a faster rate (21). Thus, a clear distinction is often made based on when the disability was acquired or developed, differentiating between early and late onset.

One of the main findings of this study is the consideration of the level of inclusion experienced by athletes, in addition to the time when the disability was acquired. The participant highlighted the inclusion and support athletes with congenital or early-acquired disabilities receive in social and educational settings:

There are people who have been ‘native inclusives’.. and then there are those who have not; I am not a ‘native inclusive’; I have had to integrate myself instead. (I).

In this way, two types of athletes with early-acquired disabilities can be differentiated: those who have been included and those who have not, a distinction that can prove crucial for their progress. The resources that athletes can draw upon to address challenges such as traumas—that is, moments in life that have negative consequences—may be decisive in their development (54). Indeed, traumas can serve as a means to acquire new skills or perspectives, or simply to develop resilience (55):

And what if I had had a different character? Perhaps we would not be talking now, and I would be a mess and a miserable person. (I).

For athletes with early-acquired disabilities, the interviewee distinguished and their previous experience with the disability in relation to potential performance. It has been noted that individuals with prior contact with sports may view the future with greater optimism than those with no previous connection, who, having recovered, need to start from scratch. Given that disabilities can be acquired over time, age is not considered a determining factor for sports success (45). It has been observed that prior sports experience is an essential component for athletes who acquire a disability later in life (21).

On the other hand, the type of sport practiced can influence an athlete's development and skill acquisition, and it may prove decisive in their sports trajectory. The nature of sport can enhance the creation of appropriate development environments for athletes, thereby affecting talent detection processes. In this regard, these environments should be as inclusive as possible (6). It has been observed how participation in sports, as in the case of blind tennis, can help people with disabilities to build a competent identity that could be transferred to their daily life (56).

I do not know if it was because they were judokas, who know that there is a learning process that takes time, and everyone assumes it is a process that takes time, which helps with not being impatient with someone’s talent. (I).

#### 3.2.3 Development strategies and recruitment of Paralympic athletes

A wide range of strategies have been implemented to attract and recruit athletes with disabilities (21). It is important to highlight the initiatives currently being developed in this area, such as sports events and competitions organized by various federations, as well as educational programs aimed at primary and secondary school students. One of the main strategies being carried out at the regional level in Spain is the program known as *Relevo Paralímpico* (“Paralympic Relay”) (57). This initiative aims to identify and locate young people with disabilities and introduce them to sports, with the goal of fostering their development to become Paralympic athletes. The program includes actions such as training technicians who provide equipment to clubs and organize sports promotion and talent detection events. According to Balyi et al., promoting Paralympic sports through the development of sports events for people with disabilities is crucial in this initial stage, as it serves a dual purpose: first, it raises awareness of sports opportunities through awareness actions (e.g., practicing a disability sport in physical education classes); and second, it provides people with disabilities with a positive initial experience in sports, which can encourage them to continue participating (58). Other initiatives are being undertaken by sports structures such as clubs and federations, as demonstrated by the events organized by the Spanish Federation of Judo previously mentioned. Foundations, as previously evidenced, also play a crucial role in this regard.

Additionally, these awareness actions are starting to be developed in the educational context, across different educational stages. A clear example is the Paralympic Day of the “Inclusive Sports in Schools” (DIE) program (59), in which the interviewee participates as a speaker. In this program, Paralympic Day is held in primary and secondary schools and is divided into two phases: (1) a disabled sports athlete visits the educational center to share their experiences and insights and exchange opinions and questions with students; and (2) a practical part where students participate in Paralympic and inclusive sports (60):

I collaborate with CEDI in primary and secondary education, offering a different perspective to all these children and removing stereotypes that affect everyone. (F); (…) I also collaborate with the DIE program so that children do not experience what I went through, to try to make the situation a little easier, to smooth the path, even though what I went through, unfortunately, still happens. (F).

Another possible entry point into sports is rehabilitation. Rehabilitation is not limited to treating injuries but can also generate interest in sports, especially for those with acquired disabilities (61):

Many athletes start doing Paralympic sports because they begin with a rehabilitation process. (I).

Significant variations exist in the methods used to detect and identify talent in the Paralympic sports field, as well as in the factors that may have a disproportionate impact on the trajectory of athletes with disabilities. One of the main differences in talent identification is that Paralympic athletes tend to be older than those without disabilities. Age is a less determining factor for sports success in this context (62), as many athletes may have faced difficulties in accessing a particular sport or have acquired a disability later in life. A clear example is the athlete interviewed, who started practicing judo at 19 and was 47 at the time of the interview. In talent identification, sports events that focus on athletes with disabilities who are already involved in sports are crucial. Thus, the Paralympic Relay program organizes two types of sports events: those aimed at sports promotion (as previously mentioned) and those focused on talent detection (57):

There were other concentrations for older athletes that were more focused on whether you like to train, on seeing where more could be extracted. (I).

However, it is equally (or even more) relevant to have the support of experts with a deep understanding of disabilities and classification systems during these events in order to support selection processes (17, 21).

At the same time, there have been attempts to implement talent detection or “talent transfer” programs between sports, where athletes have an increased opportunity to progress in a different sport (54). Coaches play a crucial role in implementing these strategies:

I think they manage among themselves because they see them, know them, call them, and maybe a kid who was playing football tells the judo coach to invite him to judo because he thinks he will do better and like it more. (I).

A lack of funding is a common factor that affects the identification and development of athletes, and sports organizations often lack the capacity to maintain effective systems due to political and financial limitations (17). Historically, Paralympic sports have received less funding compared to non-Paralympic sports, leading to a scarcity of resources and support for the development of these athletes (18, 63). This deficiency can limit the potential of Paralympic athletes to achieve their peak performance and succeed in competition. Social, economic and research limitations negatively affect the development of athletes with disabilities, restricting their opportunities to access essential resources such as coach training programs and wider sports offers, among others. On the other hand, further research in this embryonic field is necessary to offer alternative approaches to addressing these limitations:

I think scientific research could perhaps help more in terms of securing funding, obtaining laws, and achieving more tangible outcomes. (I).

It is essential to highlight the limitations of this study for a better understanding of the results obtained. The research has been conducted around a Spanish athlete, so generalizing the results and proposed strategies should be done with prudence. The choice to conduct a case study to delve deeper into this topic means that the results should be taken with caution, as they do not represent all women athletes with disabilities. Furthermore, due to the limited literature in this area, future studies should explore the various factors that influence the development of athletes with different disabilities through studies that capture the first-hand perspectives of athletes and the influence of their environment. A topic not addressed in this study is the end of the sports career of athletes with disabilities, which should be considered in future research.

## 4 Practical recommendations

It is essential to analyze the factors involved in the process of identifying athletes with disabilities who have high performance potential, as well as these individuals’ adherence to sports development programs that ensure the necessary conditions for their athletic progression in their respective sport. This case study highlights the need to treat each individual athlete independently, paying attention to their unique developmental path. Assuming that there is no single model for athletes, it is necessary to develop an environment that allows athletes to remain within the system. Therefore, the primary focus should be on facilitating the initiation and development of sports practice.

As this study has observed, the initial stages of sports participation for women with disabilities can be hampered by various barriers, most notably, a lack of role models and a lack of visibility. Due to the current scarcity of initiatives and support from sports organizations and federations, foundations play a crucial role in supporting these athletes throughout their careers. Particularly in the early and developmental stages, the coach also plays a fundamental role in retaining the athlete and addressing their progression needs.

There are several factors that can affect athletes’ performance and progress. The results of this research underscore one especially important aspect in the development of athletes with congenital or early-onset disabilities: the degree of inclusion they experienced during their early stages. Those who were not included (or whose participation was not promoted) may have developed significant resilience, which can be crucial in high-level competition; however, it is likely that many others dropped out along the way. Furthermore, the nature of the sport itself can facilitate the athlete's development, as is often seen in sports such as judo, which involves a growth process and which may promote the creation of suitable developmental environments. It is also important not to overlook the relevance of the regulations and functional classifications of each sport and specialty in athletic progress.

In these recommendations, it is pertinent to address the two specific phases of the Canadian long-term athlete development model for athletes with disabilities (64): “first involvement”, understood as the critical moment for coaches to inform, motivate, and retain potential athletes; and “awareness” of the entire sports system in favor of active participation of athletes with disabilities, by informing them of sports opportunities. Thus, education and awareness programs are essential tools in the sports development process for athletes with disabilities.

The search for athletes is a complex process influenced by various factors, including but not limited to social, economic, and political considerations. Promoting and facilitating access to sports practice from school age is crucial for this group. Initiatives such as the Paralympic Relay and Paralympic Day are highly relevant in this regard. It is necessary to implement additional activities that continue to promote sports practice and help identify potential talents. Examples include inclusive competitions organized by sports federations—where athletes with and without disabilities participate—and sports days.

## 5 Conclusions

This case study has examined the sports development of athletes with disabilities, particularly women, through the perspective of a visually impaired Paralympic athlete. First, the research has highlighted various aspects that can significantly affect these athletes’ performance, including the level of inclusion received and the specific rules of each sport. Second, it has identified different barriers that shape this group's initial stages of sports participation, notably the substantial support provided by foundations and the importance of the coach. Lastly, it has emphasized various initiatives for promoting sports in school stages (such as the Paralympic Relay and Paralympic Day) and actions aimed at fostering development and talent identification (including inclusive competitions and sports days). Nevertheless, further initiatives and increased resources are still needed in this area, particularly for women's Paralympic sports.

## Data Availability

The original contributions presented in the study are included in the article/Supplementary Material, further inquiries can be directed to the corresponding author.
